# High-risk Individuals and Naloxone Use: Implications for THN Programs in Rural Appalachian Communities

**DOI:** 10.13023/jah.0503.02

**Published:** 2023-12-01

**Authors:** Victor Garcia, Lisa McCann, Erick Lauber, Christian Vaccaro, Melissa Swauger, Alex Daniel Heckert

**Affiliations:** Prevention Research Center; Indiana University of Pennsylvania; Indiana University of Pennsylvania; Indiana University of Pennsylvania

**Keywords:** Appalachia, take-home naloxone, THN, opioid overdose, intervention, rural Appalachia, Western Pennsylvania

## Abstract

**Introduction:**

Take-home naloxone (THN) is being made available across rural Appalachia to curb opioid overdose fatalities. Despite this initiative, some opioid users do not possess naloxone, and if they do, do not administer it to others.

**Purpose:**

Research findings on risk factors that contribute to opioid overdose are presented. These factors, identified in a sample of 16 overdose cases, are (1) early onset age of opioid use; (2) progressive opioid use; (3) a transition from pain medication to heroin and fentanyl; (4) fears of being arrested at a naloxone intervention if first responders are contacted, and (5) limited knowledge of Good Samaritan Laws.

**Methods:**

The findings are based on a subsample 16 overdose victims who were identified during a one-year (2018) qualitative study on the decline of overdose fatalities in four rural counties in Western Pennsylvania. They were recruited from a larger sample of 50 current and former substance users and were interviewed a second time using a semi-structured interview guide about their overdose experiences. All interview data were analyzed using thematic analysis via NVivo.

**Results:**

Findings reveal that risk factors contribute to a severe opioid dependence that interferes with naloxone use. These factors also hinder adherence to proper naloxone protocol, designed to place overdose victims in contact with treatment providers.

**Implications:**

Recommendations are made for additional research and for pursuing measures to increase efficacy of naloxone interventions. They include developing naloxone campaigns aimed at high-risk individuals, improving their knowledge of Good Samaritan Laws, increasing adherence to THN protocols that improve the possibility of treatment, and using community harm reduction specialists for community outreach.

## INTRODUCTION

Appalachia continues to be the epicenter of the country’s opioid epidemic and has some of the highest fatal and nonfatal opioid overdose rates,^1^,^2^ especially in its rural areas.^3^,^4^ In response, the region’s communities are implementing harm-reduction measures,^5^ such as take-home naloxone (THN) distribution,^5^–^7^ as part of a broader shift from self-help traditions to evidence-based medication for opioid use disorder (MOUD) treatment strategies.

However, despite its importance for audiences within Appalachia, much THN research does not include this region or focuses narrowly on distribution issues, such as limited physician participation, lack of medical providers’ familiarity with naloxone-dispensing regulations, inability to secure naloxone supplies, and the costs involved in doing so.^8^–^11^ Recent research has examined the naloxone experiences of opioid overdose victims to better understand the impediments to THN. Much of this work, however—apart from that of Kahn and her colleagues^13^ —is not being conducted in rural Appalachia nor in other rural areas, where stigma, negative views regarding illicit substances, and self-help traditions add to the challenges facing harm-reduction programs.^16^–^18^

### Purpose

Research findings are presented on 16 people who use opioid drugs (PWODs) with risk factors that diminish their ability to possess and administer naloxone in Western Pennsylvania, within the northern region of Appalachia. The present study is descriptive in nature, and findings are grouped into the following themes: (1) participants’ knowledge of obtaining and carrying naloxone; (2) their fears about first responders when using it; (3) their past experience of its use; (4) their withdrawal symptoms from using it; and (5) how its use is decoupled from getting into treatment.

Previous research and the authors’ own data show that PWODs experience early onset age of opioid use; progressive opioid use; transition from opioid-based pain reduction medication to heroin, and in some cases from heroin to fentanyl; fears of legal consequences resulting from partaking in a naloxone intervention (i.e., administering naloxone and contacting first responders); and limited knowledge of Good Samaritan Laws). The present study examines the circumstances surrounding the individuals’ overdoses, their knowledge regarding naloxone, and their experiences with naloxone, including whether they were revived with naloxone or used it to revive someone else. Factors that prevent or facilitate naloxone use among these participants in rural areas are also identified and further explored.

## METHODS

The 16 respondents were drawn from a one-year qualitative study on the circumstances surrounding opioid overdose fatalities in 2018 in four rural counties in Pennsylvania: Armstrong, Blair, Cambria, and Indiana. All four counties were among the rural counties with the highest rates of overdose deaths in the state. The study was approved by the Institutional Review Board of Indiana University of Pennsylvania.

Fifty individuals who had previously used or who were currently using opioids were recruited using respondent- and interviewer-driven sampling. Specifically, some of our respondents were directly recruited by our interviewers, some of whom were in recovery and had contacts with current people who used drugs. Others were recruited through chain sampling. Informed consent was obtained from all participants. Twenty-seven of the 50 respondents were found to have experienced an overdose. From this subsample, 16 individuals were recruited for an additional interview using convenience sampling. Their demographic backgrounds are listed in Table 1 in the Appendix (see Additional Files).

All the current and ex-individuals who abused opioids were first interviewed face-to-face using a six-page open-ended interview guide comprised of seven sequential sections: demographic background, employment and work history, personal drug use, drug use in the community, Narcan, drug overdoses, and final comments. The subsample of 16 participants was interviewed regarding their overdose experiences, naloxone use, and treatment participation. All the respondents in the initial and second round of interviews were compensated for their time with a food gift certificate valued at $20. Interview transcriptions were coded, and the coded text was categorized and analyzed using NVivo. An inductive thematic analysis was used to examine coded text and identify themes and subthemes around overdoses, substance abuse history, naloxone experiences, and risk factors that impede naloxone use. Summaries of the identified patterns in the analysis were prepared, and de-identified interview quotes were selected to illustrate major points in these patterns.

## RESULTS

In all, there were 78 overdoses that occurred across the 16 respondents. Specifically, two participants overdosed once, six overdosed two times, three overdosed three times, two overdosed four times, and the remaining three overdosed 14, 15, and 18 times. However, only 60 of the 78 overdose episodes are reported on here. Because of potential recall problems, overdose inquiries were limited to the first eight overdoses, and the 10^th^ and last overdose if there were more than eight overdoses. Overdose and naloxone use information is listed in Table 2 in the Appendix (see Additional Files). Fourteen of the overdoses were identified as accidental; that is, the respondent did not intend to overdose. Two respondents—Participants No. 3 (PN3) and No. 14 (PN14)—identified their recent overdoses as intentional. According to them, because of the despair over their opioid dependence, they were intentionally careless in ingesting heroin or fentanyl and did not care if they overdosed and died.

All 16 participants started using illicit substances at a young age; nine began at the age of 12 or younger, and about two-thirds commenced in their teens. All but one of the respondents started their opioid use with unprescribed opioid-based pain reduction medication and progressed to heroin, fentanyl, and other opioids. Four of the 16 participants used heroin one year or less before they had their first overdose; five others between two to five years; and seven between six to eight years. Nearly all the overdoses involved injecting heroin in conjunction with using other substances. The overdoses occurred from 2005 to 2018; the majority occurred between 2014 and 2018. The respondents attributed their overdoses to the potency of the heroin dosage, fentanyl, or a suspected mixture of fentanyl with heroin, although some did not recall for some of the overdoses. In 32 of the 60 overdose episodes, the overdose victim was alone; in 20 of the episodes, they were with someone else, mainly friends, including significant others. The remainder do not recall. Most of the overdoses, 31, occurred at the respondents’ home, or at the home of their parents, another relative, or a friend. Eight took place in their personal vehicle, four in public restrooms, one in a detox center, one in a juvenile facility, and the remainder did not recall.

### Themes

#### 1. Knowing About Naloxone

Almost all of the 16 respondents knew about naloxone, but by its street name, Narcan. They associated it with preventing overdose deaths. They were familiar with naloxone because it was used to revive them, or they heard about it from other opioid users, family, and friends. Naloxone was readily available in their communities, particularly in Armstrong, Cambria, and Indiana counties, where the Single County Authority of these counties aggressively promoted its distribution. Naloxone was distributed at churches, the local university, and numerous community events. The respondents also knew that they could get naloxone from treatment providers, and their physicians and pharmacies. One participant, PN5, traded drugs for naloxone, as he shared:

*I was trading drugs for Narcan, so I always had Narcan. . . I didn’t want people dying on my hands.*

#### 2. Fearing First Responders

Despite knowledge about naloxone and the effort to distribute it, many of the participants were reluctant to carry or administer naloxone or contact 911 because of the fear or panic of being arrested and being incarcerated. PN7, who overdosed several times, shared this fear:

*When you’re with one or two people, and you guys are getting high, and somebody overdoses, people freak out. Most of the time 911 is not called, because you’re also shooting heroin with the person. People are afraid to give people help or try to give people help, because they don’t want to have a record. They don’t want to get in trouble, or they don’t want to add to their record, or they’re on probation. Then they get hit, and you gotta go sit in county again for six months.*

Some of the research participants are of the opinion that the authorities will arrest them regardless of the law. For example, as PN11 shared:

*Yeah, because I know that they passed the law that if you’re with someone when they overdose, you call and you can't get in trouble. But then, this last time I was in jail, there was three people in there that someone was with them that overdosed, and they called the ambulance, or the cops. They showed up, found drugs on him, and he ended up in jail for possession.*

PN13 shared that the law does not provide protection for those with outstanding warrants against them:

*Yeah, no doubt. And oftentimes, the people that are overdosing, they have outstanding warrants. So, although there is the Good Samaritan Law, that doesn't really apply to warrants that you have for whatever the other crimes would be.*

Furthermore, as the quote from PN11 indicates, what most of the respondents know about the Good Samaritan Law is from secondhand accounts.

#### 3. Carrying and Using Naloxone

Although respondents knew where to obtain free naloxone, only seven carried it—and even then, not always. They had naloxone not necessarily for themselves but to revive others. As PN12 told us:

*Yeah, I do. I carry Narcan with me all the time just because, even if it's not for myself, there’s so many people, especially in this area, because I know everybody, that it happens all the time. It scares the shit out of me.*

In some instances, when ingesting opioids with others, naloxone was available in the group, as PN4 shared:

*That’s another thing—most addicts that use heroin in Johnstown have that on deck cause they know it’s a very real possibility that they could fall out. So, thank God they do. . . Narcan changed the game. You think the overdose rates are high now. If it wasn't for Narcan, they'd be through the roof.*

Yet in other instances, as PN7 expressed, this was not the case:

*I’ve never been somewhere when I would be getting high and be like, “Oh. It's okay, guys. We can get high if we want. We have Narcan over here. Don't worry about that.” That doesn't happen. I think Narcan is more for people that don't shoot heroin. . . I know my parents, for example, kept Narcan in their house, because I was a piece of shit and lived at their house, and got high in their house all the time. . . I've never heard of a drug addict carrying around Narcan in case he overdoses. I don't think that happens.*

PN7 conveys what many others shared in the interviews: naloxone is not always present when ingesting opioids. The priority at the time is not pausing and checking to see if anyone has naloxone, it is on getting “high.”

Having naloxone available also involved using it to revive others. Only three of the participants used it on others. PN12 administered it more than once, as she informed us:

*There's four different people that I’ve used Narcan on. One of them, well, one was that girl at my house, I really didn't know her, but then there was one person that was a total stranger.*

Some shared a reluctance to administer it when available because they were uncertain whether the person was overdosing or just “nodding off.”

#### 4. Experiencing Naloxone Withdrawal

In all, 13 of the 16 participants have been revived from an opioid overdose with naloxone, some more than once. These individuals recalled 27 instances in which naloxone was used to revive them, mainly by a nearby friend or a relative. Because of immediate withdrawal symptoms, a consequence of opioid dependence, not all the respondents were relieved to be revived. Naloxone immediately reverses the effects of the opioid and instantly results in what is colloquially called “dope sickness.” The symptoms of dope sickness include nausea, headache, cramps, vomiting, and restlessness. PN3 said this about dope sickness:

*Cause Narcan throws you into instant withdrawal, so you’re sick as soon as you wake up. Dope sick. Every time I was dope sick, I just wanted to get high or die, or both.*

Fear of dope sickness was shared more than once, and it kept many of the overdose cases from stopping their use of opioids, despite the need to do so. PN12 shared this about his efforts:

*I couldn’t stop. Every night I would say like, “Tomorrow I'm going to try to get my shit together. I'm going to try to not get high,” and I’d wake up in the morning. I would be sick, and I’d get high just to function and do daily things.*

#### 5. Averting Treatment

Being revived with naloxone seldom resulted in treatment. Of the 26 overdose episodes that involved naloxone, only 11 resulted in treatment, and not immediately. The lack of treatment occurs because the intervention is not implemented as THN protocol calls for. EMS or other first responders are not called, or the overdose victim is not seen at a local ER. This breach does not permit what is called a “warm handoff” in which the victim is referred to a treatment provider. Without a warm handoff, it is difficult for the individual to consider treatment. Opioid dependence is powerful and keeps individuals from treatment, as PN14 shared:

*I was physically addicted to the drug. I had to have it. . . You could have the best motivational speaker in the world . . . wouldn't have stopped me. There’s nothing that could have stopped me. I believe that. Cause I’ve been pleaded with, threatened with jail time, all of that. It didn’t matter. Once I got a taste for that drug, it was on. There was no pause button. There was no halt. . . I just used until I passed out, and I woke up, and I used again until I passed out. If I wasn’t using, I was in jail. It’s that simple.*

## DISCUSSION

Like other recent qualitative studies on THN use,^12^–^17^ this study’s findings show that, despite its availability, naloxone is not always used by individuals who abuse opioids, even though they know it saves lives. A major deterrent, often alluded to—such as within Heavey et al.^12^ and Kahn et al.^13^—but not always fully discussed is severe opioid dependence, which was identified here by combining three risk factors found in the substance abuse history of the research participants. This finding is important because it suggests that THN intervention programs need to consider different substance abuse histories if they are to be effective for all individuals who are abusing opioids and in danger of overdosing.

Some studies,^14^,^15^ like the present one, also considered two other risk factors: fears of legal consequences and limited knowledge of Good Samaritan Laws. Other studies support the themes from this data analysis regarding research subjects’ awareness of THN and its availability, carrying and administering it, personal experiences with naloxone withdrawal, and averting treatment.^12^–^14^,^16^,^17^

Other themes found through this analysis highlight additional reasons for not administering a THN intervention: not wanting to disrupt someone’s high, indifference to opioid overdoses, and fear of not recognizing an overdose. These reasons mirror those found elsewhere in the literature.^12^,^14^,^15^ There is also agreement across some of the studies that being revived from an overdose is not necessarily a wake-up call for an OUD treatment.^12^,^13^,^16^ As we and Fadanelli^16^ discussed, first responders are not always contacted during a THN intervention, nor is there follow-up care of any kind. Failure to establish a warm handoff keeps the naloxone-revived individual from a medical evaluation at an ER that may result in treatment for opioid abuse and other medical conditions.

## IMPLICATIONS

These findings are informative but limited to a single rural area in Northern Appalachia. What is needed is multisite research with larger samples across the Appalachian Region that address the conditions identified in our study and the research of others. However, until then, THN programs should take immediate action and develop measures to increase the number of naloxone interventions in rural communities, where there are formidable challenges for harm-reduction programs.,^18^–^21^ Toward this end, we propose and discuss below four areas that should be considered for further attention to increase the use of THN among high-risk individuals.

### Creating Targeted/Tailored THN Campaigns

THN programs need to target high-risk individuals. Generalized THN campaigns raise general awareness in the community but do not necessarily resonate with high-risk individuals. Segmented outreach campaigns should be developed to reach these individuals. The messaging should allay their fears about initiating or participating in naloxone intervention and inform them of the state’s Good Samaritan Law. These campaigns should also include methods to reach family members of PWODs, their friends, and significant others; they are often the bystanders who not only carry naloxone but administer it.

### Considering Good Samaritan Laws

High-risk individuals and others with OUDs still fear getting arrested if they participate in a naloxone intervention, irrespective of Good Samaritan Laws. Most of their knowledge of these laws is based on secondhand accounts from friends and acquaintances who themselves may also be unfamiliar with the laws. Individuals with OUDs need to be informed about these laws from a reliable and trusted source, such as a medical or treatment provider. Good Samaritan Laws should also be part of individualized public health campaigns, just discussed, and local authorities also need to apply them in good faith. In addition, policymakers and legislators should consider making changes in the laws and regulations such that individuals are not arrested for having outstanding warrants or other reasons if acting to help others. Police should be trained and encouraged to abide by the Good Samaritan Law and its intentions.

### Developing and Improving Naloxone Interventions and Treatment

Opioid use is highly addictive with extreme physical and psychological dependence, and the longer the use, the more difficult it is to enter treatment. Missing in many of the naloxone interventions is a “warm handoff” made possible when first responders are involved. THN programs need to convey in their campaigns the need to contact first responders and to take the overdose victim to the ER during a naloxone intervention. As will be discussed next, community harm-reduction specialists may assist in community outreach and getting the word out about Good Samaritan Laws and treatment availability.

### Using Community Harm-Reduction Specialists

Medical and treatment providers need to consider using community harm-reduction specialists (CHRS) as community outreach workers. These professionals could inform PWODs about the importance of carrying and administering naloxone, reduce unwarranted fears about arrest and protections afforded through Good Samaritan Laws, and follow up a naloxone intervention with treatment. CHRSs are ideal for this kind of outreach, especially to those in long-term recovery for OUDs. They live in the same communities as the individuals who actively ingest opioids, and as such are not perceived as a threat. Their experiences and personal struggles with OUDs give them credibility among high-risk individuals, and their stories resonate with them.

**SUMMARY BOX**

**What is already known about this topic?**

The literature is replete with research findings on programmatic challenges that communities encounter in making THN available. Increasingly, there are also studies on the experiences of overdose victims with naloxone; but missing in these is an examination of the risk factors that keep individuals with OUDs from participating in a naloxone intervention.

**What is added by this report?**

This report is among the first to examine these risk factors in rural Appalachia. The risk factors identified in the present study are early onset age of opioid use; progressive opioid use; transitioning from opioid-based pain reduction medication to heroin, including from heroin to fentanyl; fears of being arrested at an overdose scene if first responders are contacted; and unfamiliarity with Good Samaritan Laws.

**What are the implications for future research?**

Findings indicate a need to research and develop measures to increase the use of naloxone among high-risk individuals in rural communities. Recommended areas for further consideration are designing naloxone campaigns aimed at these individuals, increasing knowledge of Good Samaritan Laws, improving adherence of naloxone protocols that link overdose victims to treatment, and using community harm-reduction specialists for community outreach.

## Supplementary Information


