# Supplementary material for: High-risk Individuals and Naloxone Use: Implications for THN Programs in Rural Appalachian Communities
Source: J Appalach Health. 2023 Dec 1;5(3):9–21. doi: 10.13023/jah.0503.02 (PMC11110902; doi:10.13023/jah.0503.02)
Supplement: Supplementary file 1 [file 5.3.2_Garciaetal_AdditionalFiles.docx]

**APPENDIX:** Information regarding study participants

# Table 1. Demographic Background of the 16 Research Participants

| **ID Code1** | **County** | **Age** | **Sex** | **Marital Status** | **Education** |
| --- | --- | --- | --- | --- | --- |
| PN1 | Cambria | 23 | F | Single | AA Degree |
| PN2 | Indiana | 25 | F | Single | HS Diploma |
| PN3 | Indiana | 22 | M | Single | HS Diploma |
| PN4 | Cambria | 26 | F | Single | HS Diploma |
| PN5 | Indiana | 31 | M | Single | 8^Th^ Grade |
| PN7 | Indiana | 36 | M | Single | HS Diploma |
| PN8 | Cambria | 37 | M | Engaged | 10^th^ Grade |
| PN9 | Armstrong | 38 | M | Single | MA Degree |
| PN10 | Blair | 25 | F | Single | 11^th^ Grade |
| PN11 | Armstrong | 23 | M | Single | HS Diploma |
| PN12 | Armstrong | 34 | F | Married | HS Diploma |
| PN13 | Blair | 35 | M | Single | HS Diploma |
| PN14 | Blair | 26 | M | Single | 11^th^ Grade |
| PN15 | Armstrong | 41 | F | Divorced | HS GED |
| PN16 | Armstrong | 30 | F | Single | HS Diploma |
| PN17 | Indiana | 27 | F | Single | HS Diploma |

**^1^**PN6 was omitted from the original 17 participants because we discovered during the analysis of the interview that the overdose was not the result of opioid use.

**Table 2. Overdoses, Onset Age of Substance and Opioid Use, Opioid Use History, and Naloxone Experiences of the Research Participants**

| **ID**  **Code^1^** | **Sex** | **Age** | **No. Overdoses** | **Onset age of substance use**  **(years)** | **Onset age of opioid use (years)** | **Opioid History^2^** | **Revived with naloxone?** | **Revived others with naloxone?** |
| --- | --- | --- | --- | --- | --- | --- | --- | --- |
| PN1 | F | 23 | 4 | 12 | 14 | Pain med.**^3^**, heroin, Suboxone | Yes | Yes |
| PN2 | F | 25 | 3 | 12 | 15 | Pain med., heroin, Transdermal fentanyl | Yes | No |
| PN3 | M | 18 | 18 | 12 | 16 | Heroin, pain med., Transdermal fentanyl | Yes | Yes |
| PN4 | F | 26 | 1 | 10 | 17 | Pain med., heroin, Suboxone, Methadone, Transdermal fentanyl | Yes | No |
| PN5 | M | 31 | 3 | 7 | 15 | Pain med., heroin, U- 47700 | Yes | Yes |
| PN7 | M | 36 | 15 | 13 | 18 | Pain med., heroin | Yes | No |
| PN8 | M | 37 | 4 | 16 | 17 | Pain med., heroin | Yes | No |
| PN9 | M | 38 | 2 | 10 | 19 | Pain med., heroin, Fentanyl | Yes | No |
| PN10 | F | 25 | 3 | 14 | 18 | Heroin, pain med. | No | Yes |
| PN11 | M | 23 | 2 | 12 | 15 | Pain med., heroin | Yes | No |
| PN12 | F | 34 | 2 | 11 | 16 | Pain med., heroin | Yes | No |
| PN13 | M | 35 | 2 | 14 | 16 | Pain med., heroin, Methadone | Yes | No |
| PN14 | M | 26 | >14 | 14 | 16 | Pain med., heroin | Yes | No |
| PN15 | F | 41 | 1 | 11 | 18 | Heroin, Methadone | No | No |
| PN16 | M | 30 | 2 | 8 | 18 | Pain med., heroin | No | No |
| PN17 | F | 27 | 2 | 15 | 15 | Pain med., Transdermal fentanyl, heroin | Yes | Yes |

**^1^** PN6 was omitted from the initial 17 participants because we discovered during the analysis of the interview that the overdose was not the result of opioid use.

**^2^** The opioids in the “Opioid History” column are listed in the order that the respondents began to use them. Suboxone and Methadone are listed because they were being used without a prescription from a medical provider*.*

**^3^** “Pain med” is in reference to unprescribed opioid-based pain reduction medication, mainly in pill form.
